# Factors correlated with excessive daytime sleepiness in patients with Parkinson's disease: A polysomnography study

**DOI:** 10.1002/brb3.3202

**Published:** 2023-08-02

**Authors:** Yuan Shen, Haicun Shi, JianGuo Zhong, PingLei Pan, ShuFang Wang, MingZhu Chen, ZhiPeng Chen, ChunFeng Liu

**Affiliations:** ^1^ Department of Neurology The Yancheng School of Clinical Medicine of Nanjing Medical University, The Sixth Affiliated Hospital of Nantong University, Yancheng Third People's Hospital Yancheng China; ^2^ Department of Neurology The Second Affiliated Hospital of Soochow University Suzhou China

**Keywords:** correlative factors, excessive daytime sleepiness, Parkinson's disease, polysomnography

## Abstract

**Objective:**

To explore the factors correlated with excessive daytime sleepiness (EDS) in patients with Parkinson's disease (PD).

**Methods:**

A total of 239 PD patients were divided into two groups based on the presence of EDS (Epworth Sleepiness Scale score≥10) (PD‐EDS vs. PD‐non‐EDS). Participants underwent an extensive assessment to determine demographic features, disease severity, polysomnography characteristics, and nonmotor symptoms.

**Results:**

Of the 239 patients, 56 patients (23.4%) were classified as having PD combined with EDS. Binary logistic regression analysis showed that fatigue (Fatigue Severity Scale [FSS] score ≥4) (odds ratio [OR] [95% CI] = 4.897 [2.376–10.095], *p* < .001) and the respiratory‐related microarousal index (OR [95% CI] = 2.063 [1.085–3.923], *p* = .027) were independent risk factors for EDS in PD patients. A priori‐determined stratified analysis showed that after adjustment for confounding factors, the association of the respiratory‐related microarousal index with EDS was significant (OR = 4.404, 95% CI 1.673–11.592, *p* trend = .036) in patients with respiratory arousal index scores in the highest quintile compared with those with scores in the lowest quintile.

**Conclusion:**

Our data revealed a close association among the respiratory‐related microarousal index, FSS scores, and EDS. It can be speculated that fragmented sleep and pathological abnormalities of the central nervous system resulting in changes in arousal are major influencing factors of EDS in PD.

## Graphical abstract

Our data revealed a close association between the respiratory‐related microarousal index, FSS scores and EDS. It can be speculated that fragmented sleep and pathological abnormalities of the central nervous system with changes in arousal were major influencing factors of EDS in PD.

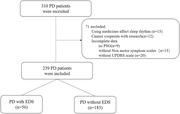

## 1 INTRODUCTION

Research developments have shown that Parkinson's disease (PD) is not only heterogeneous in terms of motor disorders but also has a variety of nonmotor symptoms (NMSs), including sleep disorders, cognitive dysfunction, sensory disorders, and autonomic nervous dysfunction. NMSs of PD can have a serious negative impact on the ability of daily living and the quality of life of patients; these symptoms can even have a stronger impact on patients’ social resilience than movement disorders. Sleep disorders, such as excessive daytime sleepiness (EDS), insomnia, rapid eye movement sleep behavior disorder (RBD), sleep‐disordered breathing (SDB), restless leg syndrome, and periodic leg movement, are common NMSs of PD with an estimated prevalence of 9% to more than 83% (Maggi et al., 2023).

EDS is defined as the inability to remain awake during the usual waking hours of the day due to uncontrollable sleep needs (Sateia, 2014). It is one of the most common sleep disorders among PD patients, affecting 21%−76% of PD patients (Loddo et al., 2017). A recent meta‐analysis on the prevalence and clinical aspects of EDS showed that the pooled prevalence of EDS was 35% in PD patients (Maggi et al., 2023). It has a significant negative impact on quality of life and driving safety. Therefore, it is essential to fully understand the neurobiological mechanism underlying this symptom. The exact pathophysiology of EDS in PD remains largely unknown.

Previous studies have shown that EDS is most commonly a result of insufficient sleep. In addition, EDS is associated with sleep apnea, fatigue, and circadian symptom rhythm sleep‐wake disorders (Guilleminault & Brooks, 2001). Of these factors, fatigue is difficult to differentiate from EDS. Fatigue usually refers to feelings of tiredness at rest, a lack of energy that compromises daily activities, or even loss of vigor (Nassif & Pereira, 2018). The concept of fatigue has always been difficult to define. It is important for medical personnel to understand that fatigue and sleepiness are not synonymous. Although a large number of publications have demonstrated an increasing interest in clinical practice and research on fatigue, fatigue is still underestimated (Maestri et al., 2020). Previous studies have shown that fatigue may be related to other NMSs, such as EDS (Alves et al., 2004; Chung et al., 2013), but they are not always associated; moreover, fatigue may occur in isolation. However, EDS cannot be explained by this comorbidity alone. In more than half of patients, fatigue is persistent and seems to be an independent symptom (Valko et al., 2010).

In the general population, EDS is a major limiting symptom of obstructive sleep apnea, which affects daytime functioning and quality of life (Ulander et al., 2022), but the specific mechanism is not yet clear (Tomfohr et al., 2011). There is still controversy over whether PD patients with obstructive sleep apnea syndrome can experience EDS, and the sample size involved is relatively small (Poryazova et al., 2010; Sobreira‐Neto et al., 2019). To date, research on the impact of sleep microstructure on EDS in PD patients is very limited (Schreiner et al., 2023).

Currently, treatment options for PD‐related sleep disorders are very limited, and alertness difficulties have a negative influence. Therefore, it is particularly important to explore the risk factors for EDS to improve its prevention.

## 2 METHODS

### 2.1 Subjects

This was a retrospective study. All data were collected from PD patients admitted to the Department of Neurology in the Second Affiliated Hospital of Soochow University from September 2013 to July 2017.

#### 2.1.1 Inclusion criteria

The inclusion criteria were as follows: (1) diagnosis of idiopathic PD based on the United Kingdom Parkinson's Disease Society Brain Bank criteria (Hughes et al., 1992) and (2) older than 18 years of age.

#### 2.1.2 Exclusion criteria

The exclusion criteria were as follows: (1) secondary Parkinson's syndrome; (2) hypophonia occurring in PD, mental illness, or other reasons for an inability to cooperate with PD severity assessment or neuropsychological assessment; (3) a history of drug and alcohol abuse; (4) a history of using medicines known to affect melatonin secretion, such as exogenous melatonin and melatonin receptor agonist; (5) shift work, which is a recurring work schedule that overlaps the usual time designated for sleeping, including night‐shift work, defined as having ever worked for at least 3 h between midnight and 5:00 am, and early‐morning shifts, defined as starting work after 2:00 AM but before 6:00 AM at least three times per month for at least 1 year (Barul et al., 2019); and (6) incomplete clinical data, laboratory data, and polysomnography (PSG) data.

### 2.2 Study protocol

#### 2.2.1 Demographics, detailed clinical presentation, and history

The following information was collected from all patients: demographic characteristics, body mass index, education, time of onset, PD duration, treatment, and medication history. The levodopa equivalent dose (LED) was calculated by using the unified formula (Tomlinson et al., 2010) with additional consideration of opicapone (levodopa dose *0.5) and safinamide (LED = 100 mg) intake (Schade et al., 2020). The Unified Parkinson's Disease Rating Scale motor examination (UPDRS‐III), Hoehn and Yahr stage, and NMS questionnaires were administered to all patients. The NMS questionnaires specifically included the Epworth Sleepiness Scale (ESS), Pittsburgh Sleep Quality Index (PSQI), Fatigue Severity Scale (FSS), Hamilton Depression Scale (HAMD), and Hamilton Anxiety Scale (HAMA), which were self‐report questionnaires. UPDRS‐III, Hoehn and Yahr stage, and Montreal Cognitive Assessment (MoCA) were assessed by two neurologists specializing in movement disorders.

The MoCA was used to evaluate the degree of cognitive dysfunction in PD patients. The maximum score is 30. MOCA scores < 24 are considered cognitive impairment. If the subject had less than 12 years of education, one point was added to the original score (Tsai et al., 2016).

The HAMA and HAMD were used to evaluate the degree of mood disorders in PD patients. A score of HAMA≥7 was defined as having anxiety, and a score of HAMD≥8 was defined as having depression.

The ESS and the PSQI were used to assess the degree of sleep disturbance in PD patients. ESS assessed the daytime sleepiness of patients, with a total score of 0–14; scores of 7–9 were considered to indicate suspicion of daytime sleepiness, and scores ≥10 were considered to indicate EDS (Sander et al., 2016). The PSQI was used to evaluate the sleep quality of subjects in the last month, with a total score of 0–21. Scores ≥7 indicated the existence of sleep disorders. The higher the score, the worse the sleep quality was (Buysse et al., 1989).

The FSS was used to evaluate the severity of feelings of fatigue. The FSS has a total of 9 items, and fatigue was identified when the total score of the 9 items ÷ 9 was > 4 (Learmonth et al., 2013).

### 2.3 PSG recordings

All subjects underwent one‐night audio and video PSG in the sleep unit. PSG was performed with monitoring equipment (Compumedics‐E series), and sleep recording included electroencephalogram (F3‐A2, F4‐A1, C3‐A2, C4‐A1, O1‐A2, and O2‐A1), electrooculogram (EOG, LOC‐A2, and ROC‐A1), electromyography of the chin and bilateral anterior tibial muscles, electrocardiography, nasal airflow pressure measurement, thermal oronasal airflow, thoracic and abdominal respiratory efforts, oxygen saturation, snoring sound, and body position. Sleep stages, spontaneous microarousal, respiratory‐related microarousal, apnea−hypopnea index (AHI), periodic leg movements during sleep, and rapid eye movement（REM） sleep muscular tone were scored by visual inspection according to the American Academy of Sleep Medicine recommendations (Berry et al., 2012; Iber et al., 2007). The tonic chin electromyography activity density and the phasic chin electromyography activity density during REM sleep were calculated using the methods in PSG Diagnostic Criteria for RBD in 2010 (Montplaisir et al., 2010). According to the International Classification of Sleep Disorders criteria, RBD was diagnosed using PSG and clinical evaluations. The definitions of obstructive sleep apnea (OSA) are as follows: Apnea: Oral and nasal airflow stopped for ≥ 10 s while chest and abdominal movements continued was considered obstructive apnea. Hypopnea: Score a respiratory event as a hypopnea if all of the following are met: (1) The peak signal excursions drop by ≥ 30% of pre‐event baseline using nasal pressure (diagnostic study),positive airway pressure(PAP) device flow (titration study), or an alternative hypopnea sensor. (2) The duration of the ≥ 30% drop in signal excursions is ≥ 10 s. (3) There is ≥ 3% oxygen desaturation from pre‐event baseline or the event is associated with an arousal. AHI: The average number of obstructive apnea and hypopnea events per hour during sleep. In this study, OSA was defined as AHI≥ 5, with typical symptoms such as nocturnal sleep snoring accompanied by apnea and daytime drowsiness, according to the international standard of OSA. All PSG parameters were scored by a PSG technologist who was blinded to the clinical status and questionnaire data.

### 2.4 Statistical analysis

SPSS for Windows, version 23.0 (IBM Corporation), was used for statistical analysis. Categorical variables are presented as percentages (%), and quantitative variables are presented as the mean ± standard deviation (SD) or median (interquartile range) according to the normality of their distribution. The chi‐square test was used for categorical data. Normally distributed variables were compared using an independent samples *t* test. Continuous nonnormally distributed variables were compared using the Mann‒Whitney U test. Binary logistic regression was used for multivariate analysis of the baseline characteristics identified as risk factors for EDS in the univariate analyses, and *p* < .05 was used as the cutoff for inclusion. Binary logistic regression was used to estimate the odds ratio (OR) of EDS by respiratory‐related microarousal stratified into five categories. A test for linear trend was conducted using quantiles of respiratory‐related microarousal level variables as continuous variables by assigning the median values of the quintiles to the variables.

All statistical tests were two‐sided, and a *p* value < .05 was considered statistically significant.

## 3 RESULTS

**FIGURE 1:**
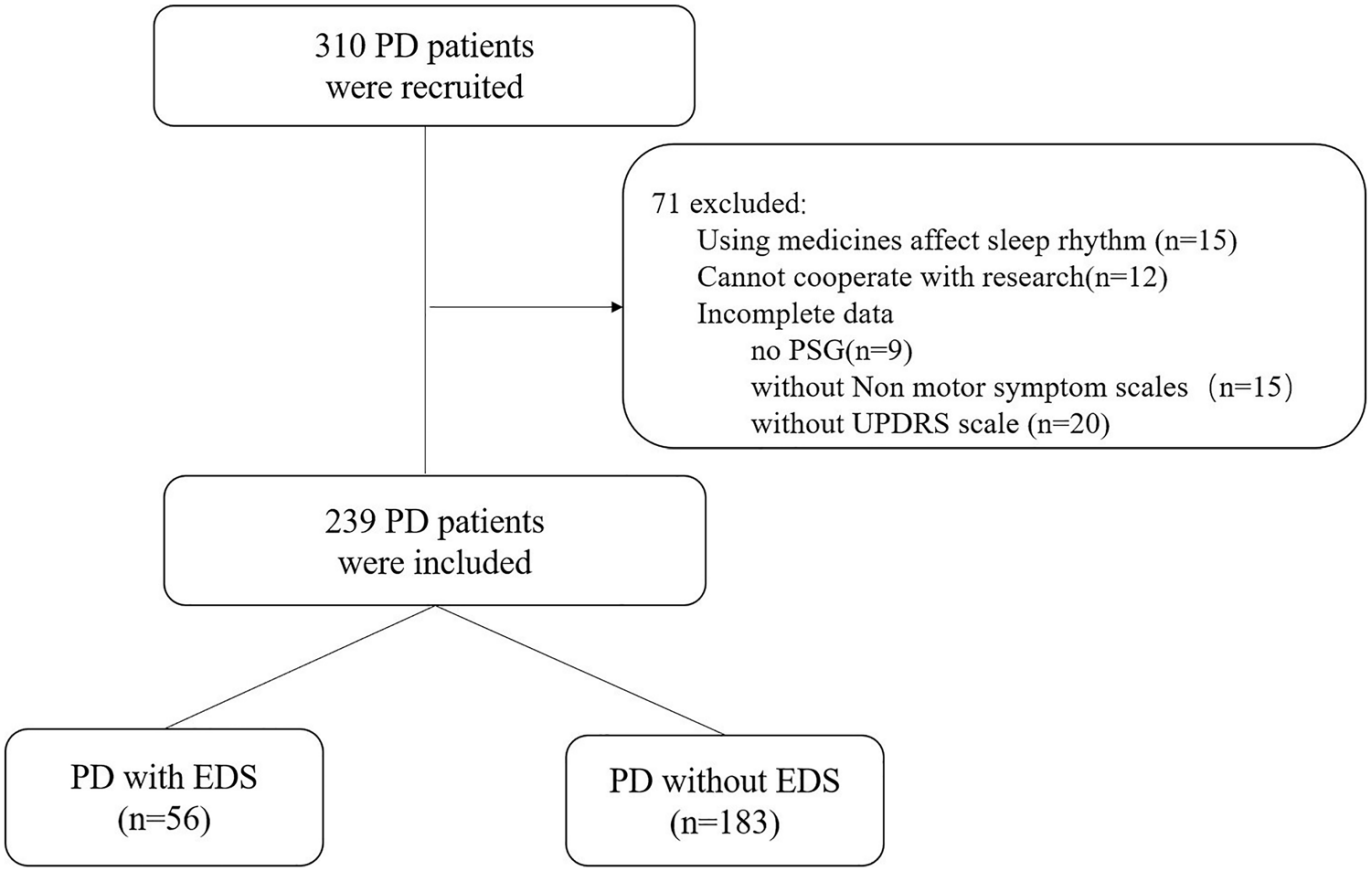
Flow diagram for patient recruitment. EDS, excessive daytime sleepiness; PD, Parkinson's disease; PSG, polysomnography.

A total of 310 patients were recruited in the study. Of these, 239 patients were finally included. The reasons for exclusion are shown in Figure 1. Of all the participants, 56 patients (23.4%) were classified as having PD combined with EDS, and 183 patients (76.6%) were classified as having PD without EDS.

1. Comparisons of demographic data and clinical characteristics between the PD‐non‐EDS and PD‐EDS groups

Comparisons of the demographic data and clinical characteristics between the PD‐non‐EDS and PD‐EDS groups are summarized in Table 1. PD‐EDS patients had a higher H‐Y stage than PD‐non‐EDS patients (2.50 [2.00, 2.88] vs. 2.00 [1.50, 2.50], *p* = .038).

1. Comparisons of NMSs between the PD‐non‐EDS and PD‐EDS groups

**TABLE 1:** Comparison of demographics and basic disease characteristics between PD patients with and without EDS.

Characteristics	PD+EDS (*n* = 56)	PD+non‐EDS (*n* = 183)	*p*
Age (years)	67.00 (61.25, 72.75)	65.00 (56.00, 72.00)	.190
Age≥65 years, *n* (%)	33 (58.9)	93 (50.8)	.288
Gender (male), *n* (%)	38 (67.9)	119 (65.0)	.696
Duration (years)	4 (2, 8)	4 (1.5, 6)	.266
Duration≥5 years, *n* (%)	22 (39.3)	58 (31.7)	.292
H‐Y stage	2.50 (2.00, 2.88)	2.00 (1.50, 2.50)	**.038**
H‐Y stage≥3, *n* (%)	14 (25)	34 (18.6)	.294
UPDRSIII	23 (15, 30.75)	22 (15, 29)	.271
Education (years)	9 (9, 12)	9 (5, 12)	.408
Education≥12, *n* (%)	22 (39.3)	74 (40.4)	.878
LED (mg/day)	300 (0, 500)	200 (0, 450)	.392
LED≥500 mg/day, *n* (%)	17 (30.4)	45 (24.6)	.389

Abbreviations: EDS, excessive daytime sleepiness; LED, levodopa equivalent dose.

Comparisons of NMSs between the PD‐non‐EDS and PD‐EDS groups are summarized in Table 2. PD‐EDS patients had higher PSQI and FSS scores than PD‐non‐EDS patients (9.00 [6.00, 11.75] vs. 7.00 [5.00, 11.00], *p* = .026; 3.00 [1.82, 4.00] vs. 2.00 [1.00, 3.00], *p* < .001) and higher proportions of PSQI scores ≥7 and FSS scores ≥4 than PD‐non‐EDS patients (40 [71.4%] vs. 103 [56.3], *p* = .043; 21 [37.5] vs. 22 [12.0], *p* < .001).

1. Comparisons of the PSG parameters between the PD‐non‐EDS and PD‐EDS patients.

**TABLE 2:** Comparison of NMS between PD patients with and without EDS.

Characteristics	PD+EDS (*n* = 56)	PD+non‐EDS (*n* = 183)	*p*
PSQI score	9.00 (6.00, 11.75)	7.00 (5.00, 11.00)	**.026**
PSQI score≥7, *n* (%)	40 (71.4)	103 (56.3)	**.043**
FSS score	3.00 (1.82, 4.00)	2.00 (1.00, 3.00)	**<.001**
FSS score≥4, *n* (%)	21 (37.5)	22 (12.0)	**<.001**
HAMD score	6.50 (3.25, 10.75)	6.00 (3.00, 14.00)	.842
HAMD score≥8, *n* (%)	22 (39.2)	78 (42.6)	.658
HAMA score	8.00 (4.00, 12.00)	8.00 (5.00, 11.00)	.965
HAMA score≥7, *n* (%)	29 (51.8)	95 (51.9)	.987
MoCA score	24.00 (23.00, 26.00)	24.00 (20.00, 26.00)	.447
MoCA score < 24, *n* (%)	17 (30.4)	82 (44.8)	.055

Abbreviations: FSS, Fatigue Severity Scale; HAMA, Hamilton Anxiety Scale; HAMD, Hamilton Depression Scale; MoCA, Montreal Cognitive Assessment; PSQI, Pittsburgh Sleep Quality Index.

Comparisons of the PSG parameters between the PD‐non‐EDS and PD‐EDS patients are summarized in Table 3. Compared to PD‐non‐EDS patients, PD‐EDS patients exhibited a lower percentage of stage N2 (43.10 [30.90, 53.38] vs. 48.50 [38.10, 59.10], *p* = .047) and had higher AHI scores (2.00 [0, 8.73] vs. 0.70 [0, 4.90], *p* = .033), respiratory arousal index scores (0.30 [0, 2.10] vs. 0 [0, 0.60], *p* = .015), and O_2_ desaturation index values (2.35 [0.50, 6.85] vs. 0.90 [0, 4.90], *p* = .011).

1. Multivariable analysis

**TABLE 3:** Comparison of sleep parameters between PD patients with and without EDS.

Characteristics	PD+EDS (*n* = 56)	PD+non‐EDS (*n* = 183)	*p*
TST, min	314.32 ± 92.57	335.74 ± 110.48	.190
SE, %	61.90 (48.58, 75.35)	67.40 (51.20, 77.60)	.295
SL, min	11.50 (4.13, 21.25)	14.50 (5.00, 33.00)	.198
WOAS, min	21.50 (16.00, 30.75)	20.00 (15.00, 29.00)	.215
Stage N1 (%)	27.87 ± 17.47	23.67 ± 15.47	.086
Stage N2 (%)	43.10 (30.90, 53.38)	48.50 (38.10, 59.10)	**.047**
Stage N3 (%)	13.45 (3.45, 23.18)	12.90 (2.90, 20.30)	.667
REM sleep latency (min)	156.97 ± 106.02	158.93 ± 115.69	.910
REM sleep (%)	15.10 (7.40, 19.70)	13.65 (8.40, 19.10)	.632
Oxygen reduction index	2.35 (0.50, 6.85)	0.90 (0, 4.90)	**.011**
Time SaO 2 < 90% (% of TST)	0 (0, 0.60)	0 (0, 0.10)	.229
Nadir SaO 2	90.50 (88.00, 93.00)	92.00 (89.00, 93.00)	.193
AHI	2.00 (0, 8.73)	0.70 (0, 4.90)	**.033**
Respiratory arousal index (/h)	0.30 (0, 2.10)	0 (0, 0.60)	**.015**
Spontaneous arousal index (/h)	4.40 (2.63, 9.25)	4.50 (1.80, 8.30)	.625
PLMS	26.00 (0.65, 53.65)	8.80 (0.80, 40.05)	.113
RBD, *N* (%)	29 (51.8)	86 (47.0)	.530
Phasic RWA (%)	14.10 (5.38, 43.47)	15.76 (4.95, 33.30)	.480
Tonic RWA (%)	8.04 (1.49, 26.67)	7.41 (1.35, 20.37)	.799

Abbreviations: AHI, apnea−hypopnea index; PLMS, periodic limb movement in sleep; RBD, REM sleep behavior disorder; REM, rapid eye movement; RWA, REM sleep muscle atonia; SaO2, arterial oxygen saturation; SE, sleep efficiency; SL, sleep latency; TST, total sleep time; WASO, wake after sleep onset.

The independent variables with *p* < .05 in univariate analysis were included in the binary regression equation. Binary logistic regression analysis showed that FSS (OR [95% CI] = 4.897 [2.376–10.095], *p* < .001) and respiratory‐related microarousal index (OR [95% CI] = 2.063 [1.085–3.923], *p* = .027) were independent risk factors for EDS in PD patients (Table 4).

**TABLE 4:** Binary logistic regression model examining predictors of EDS in PD.

Variable	Beta coefficient	Standard error	Wald	*p* Value	OR	95% CI
FSS≥4	1.589	0.369	18.528	<.001	4.897	2.376–10.095
Respiratory arousal index	0.724	0.328	4.874	.027	2.063	1.085–3.923

*Note*: The independent variables with *p* < .05 in univariate analysis were included in the binary regression equation.

Abbreviation: FSS, Fatigue Severity Scale.

In a priori‐determined stratified analysis, we found that there were no associations between the respiratory‐related microarousal index and EDS after adjusting for H‐Y stage (OR = 2.961, 95% CI 1.215–7.220, *p* trend = .200) and after further adjustment for PSQI and FSS (OR = 4.005, 95% CI 1.547–10.367, *p* trend = .058). After further adjustment for N2% and AHI, the association of the respiratory‐related microarousal index with EDS was significant (OR = 4.404, 95% CI 1.673–11.592, *p* trend = .036) in patients with respiratory arousal index values in the highest quintile compared with patients with values in the lowest quintile (Table 5).

**TABLE 5:** ORs (and 95% CIs) of EDS by quintiles of respiratory arousal index.

Variable	1	2	3	4	5	*p* Trend
Respiratory arousal index						
Median	0	0.2	0.4	1.55	10	
Model 1	1.00 (ref)	1.386 (0.411–4.677)	1.340 (0.539–3.332)	1.666 (0.658–4.220)	2.961 (1.215–7.220)	.200
Model 2	1.00 (ref)	1.841 (0.514–6.595)	1.189 (0.452–3.129)	2.216 (0.825–5.948)	4.005 (1.547–10.367)	.058
Model 3	1.00 (ref)	2.332 (0.644–8.443)	1.157 (0.440–3.043)	2.271 (0.848–6.084)	4.404 (1.673–11.592)	.036

*Note*: ORs and 95% CIs were calculated with the use of the binary logistic regression model.

Test for trend based on variable containing median value for each quintile.

Model 1 was adjusted for Hoehn & Yahr stage.

Model 2 was adjusted for Hoehn & Yahr stage, PSQI, and FSS.

Model 3 was adjusted for Hoehn & Yahr stage, PSQI, FSS, N2%, and AHI.

## 4 DISCUSSION

In this study, we found that the prevalence of EDS (ESS ≥10) among PD patients in our cohort was 23.4%. We found that among PD patients, EDS was related to FSS and respiratory arousal index scores independent of the H‐Y stage, PSQI, N2%, and AHI.

EDS is one of the most common NMSs of PD, affecting 21%−76% of PD patients (Loddo et al., 2017). In the general population, the prevalence of EDS is 16%−19% (Arnulf, 2005; Stavitsky et al., 2010). The wide range in prevalence rates in PD patients may be due to differences in selection bias or genetic and environmental factors. The meta‐analysis found that the prevalence of EDS in PD varied among geographic regions. Patients residing in South and North America, Europe, and Australia had a higher prevalence of EDS than those residing in Asia (Feng et al., 2021).

EDS is common in PD, and the severity of the disease, “wearing off,” and SDB have been shown to affect PD‐related EDS (Poryazova et al., 2010). There was a slight correlation between EDS and Hoehn and Yahr scores in some previous studies (O'Suilleabhain & Dewey, 2002; Pal et al., 2001) but not all studies (Arnulf et al., 2002; Razmy et al., 2004). Our study demonstrated a higher H‐Y stage in individuals with EDS than in those without EDS. However, multivariate regression analysis showed that H‐Y was not an independent risk factor for EDS after adjusting for other confounding factors. This is consistent with previous research results.

However, after adjusting for other confounding factors, FSS scores were still independently associated with EDS in PD patients. Previous studies have shown that EDS is associated with other sleep disorders, fatigue, apathy, anxiety, and other NMSs but not with motor symptoms. Valko et al. (2010) found that 48% of PD patients had EDS, 59% of PD patients had fatigue, and 35% of PD patients had NMSs at the same time. In a community‐based study, including 233 patients with PD, Alves et al. (2004) found that fatigue was associated with EDS, although the prevalence of fatigue remained high when patients with EDS were excluded, suggesting that fatigue may be caused by sleepiness. In contrast, Havlikova et al.’s (2008) study showed that sleepiness did not show a significant association with fatigue in any of the fatigue domains. There is increasing evidence that sleepiness and fatigue arise from distinct neural mechanisms. Yousaf et al. (2018) combined [123I] FP‐CIT‐SPECT with MR‐based structural imaging and demonstrated a loss of dopaminergic function in the caudate nucleus in PD patients with EDS compared to patients without EDS. The caudate nucleus is a major receptive component of the basal ganglia embedded in a large neural network. The striatum receives afferent fibers from different cortical regions, projects them to the thalamus and brain stem, and transmits them back to the cortex through the pallidum. The caudate nucleus is involved in promoting wakefulness, while the nucleus accumbens mainly enhances sleepiness. Loss of dopamine in the caudate body may actually lead to sleepiness in PD patients (Yousaf et al., 2018). Other studies have shown that EDS may be related to more significant neurodegeneration within the ascending arousal system of the brainstem, implicating the involvement of nondopaminergic mechanisms (Amara et al., 2017; Arnulf et al., 2015). There is preliminary evidence that fatigue may be related to frontal executive dysfunction. The exact relationship between central nervous system pathology and PD fatigue is still unclear. Chaudhuri and Behan (2004) proposed a general fatigue model caused by the central nervous system, where it is assumed that dysfunction of the circuits connecting the basal ganglia and the medial frontal area (such as the anterior cingulate gyrus) can lead to fatigue. Some studies have found that PD fatigue is associated with low prefrontal perfusion in SPECT and the performance of Wisconsin card sorting tasks. Another study found that the 5‐HT dysfunction of the striatum and insula in patients with PD fatigue supports the nondopaminergic mechanism and extends the potential related dysfunction to the marginal and reward regions (Herlofson & Kluger, 2017). Therefore, EDS and fatigue are independent of each other, and they are associated with different parts of the central nervous system, but the mechanisms underlying the development of EDS and fatigue may share an overlapping nondopaminergic substrate.

Although sleep fragmentation and lack of sleep are the main determinants of EDS in normal people and people with other clinical diseases, there are few records of nocturnal objective PSG parameters other than multiple sleep latency test(MSLT) in EDS studies. In a study by Ferreira et al. (2006), the PSQI was used to evaluate the subjective sleep quality of patients. There was no difference in sleep quality between patients with and without EDS, and there was no correlation between ESS and PSQI scores. Moreover, Rye et al. (2000) found that the severity of EDS was positively correlated with the prolongation of total sleep time, shortened sleep latency, increased sleep efficiency, and shortened light sleep time in a series of unselected patients with PD. Some studies have shown that there is no correlation between EDS and objective sleep parameters, including total sleep time, sleep efficiency, sleep latency, and slow wave sleep time (Liguori et al., 2019; Stauder et al., 2022).

In our study, compared with patients without EDS, PD patients with EDS had a lower percentage of N2 sleep and higher PSQI scores. However, after adjusting for other confounding factors, there was no independent correlation between EDS and either of these factors. This is consistent with previous studies (Arnulf et al., 2002).

There are few studies on the relationship between EDS and respiratory events in patients with PD, and the existing research conclusions are inconsistent (Suzuki et al., 2013). According to the research by Suzuki et al. (2013), OSA was not the primary cause of EDS in PD, and EDS was more likely due to other pathologic factors associated with PD. Cochen De Cock et al. (2010) compared a group of PD subjects with EDS to a group of PD subjects without EDS and did not record any difference in the prevalence of OSA between the two groups. Sobreira‐Neto et al. (2019) also showed that there was no difference in the prevalence of OSA in PD patients with and without EDS, which strengthened the view that OSA had little effect on the existence of EDS. In contrast, Shpirer et al. (2006) observed that the average ESS score of PD patients with OSA was higher than that of patients without OSA. Through a randomized, double‐blind study, Neikrug et al. (2014) recorded the improvement of ESS in a group of PD patients who began to use a positive pressure device to treat OSA, indirectly reflecting the influence of OSA on EDS in PD patients. In our study, univariate analysis showed that the oxygen deficit index, AHI, and respiratory‐related microarousal index of PD patients with EDS were higher than those of patients without EDS, but in the multivariate analysis, after adjusting for other confounding factors, only the respiratory‐related microarousal index was independently correlated with EDS in PD patients. It has been confirmed that nocturnal hypoxemia plays little role in the pathogenesis of daytime sleepiness in OSA patients. Oxygen therapy may improve nocturnal oxygen saturation in OSA patients, but it does not improve sleep latency as measured by MSLT. In contrast, the sleep segments formed by nonrespiratory stimulation were not accompanied by nocturnal hypoxemia but could still lead to severe daytime sleepiness in the general population (Colt et al., 1991). It has been suggested that hypoxia may lead to an inflammatory response, but it will not cause daytime sleepiness (Burioka et al., 2009).

A major limitation of our study is that it was a retrospective analysis, and thus, there might be a selectivity bias. Second, there was no EDS group or normal control group in the study, which limited our ability to compare the results with those in the general population. In addition, EDS was assessed using only the subjective ESS, not the objective MSLT test. However, objective and subjective EDS results are often disparate in PD (Cochen De Cock et al., 2014). However, subjective EDS consistently shows significant clinical outcomes and reduced quality of life and is a useful research tool for prognostic stratification. Finally, as a single‐center study, a referral bias cannot be ruled out.

In summary, our data showed a close association among respiratory‐related microarousal, FSS scores, and EDS. It can be speculated that fragmented sleep and pathological abnormalities of the central nervous system with changes in arousal were major influencing factors of EDS in PD.

## AUTHOR CONTRIBUTIONS

**Yuan Shen**: Data collection, statistical analysis, and article writing. **Haicun Shi**: Article review. **JianGuo Zhong**: Project design, review articles. **PingLei Pan**: Article writing guidance. **ShuFang Wang, MingZhu Chen, ZhiPeng Chen**: Collecting data. **ChunFeng Liu**: Project design, review article.

## CONFLICT OF INTEREST STATEMENT

No conflict of interest need to be declared by the authors.

### PEER REVIEW

The peer review history for this article is available at https://publons.com/publon/10.1002/brb3.3202.

## ETHICS STATEMENT

The study was approved by the Second Affiliated Hospital of Soochow University's ethical committee. All participants provided written informed consent to participate.

## Data Availability

The datasets used and/or analyzed during the current study are available from the corresponding author on reasonable request.
